# Supplementary material for: A Scoping Review of the Global Distribution of Causes and Syndromes Associated with Mid- to Late-Term Pregnancy Loss in Horses between 1960 and 2020
Source: Vet Sci. 2022 Apr 13;9(4):186. doi: 10.3390/vetsci9040186 (PMC9032147; doi:10.3390/vetsci9040186)
Supplement: Supplementary file 1 [file vetsci-09-00186-s001.zip › vetsci-1641922-supplementary.pdf]

A scoping review of global distribution of causes and syndromes associated with mid to late-term pregnancy loss in horses between 1960 and 2020: Supplementary Material from the Multiple Correspondence Analysis

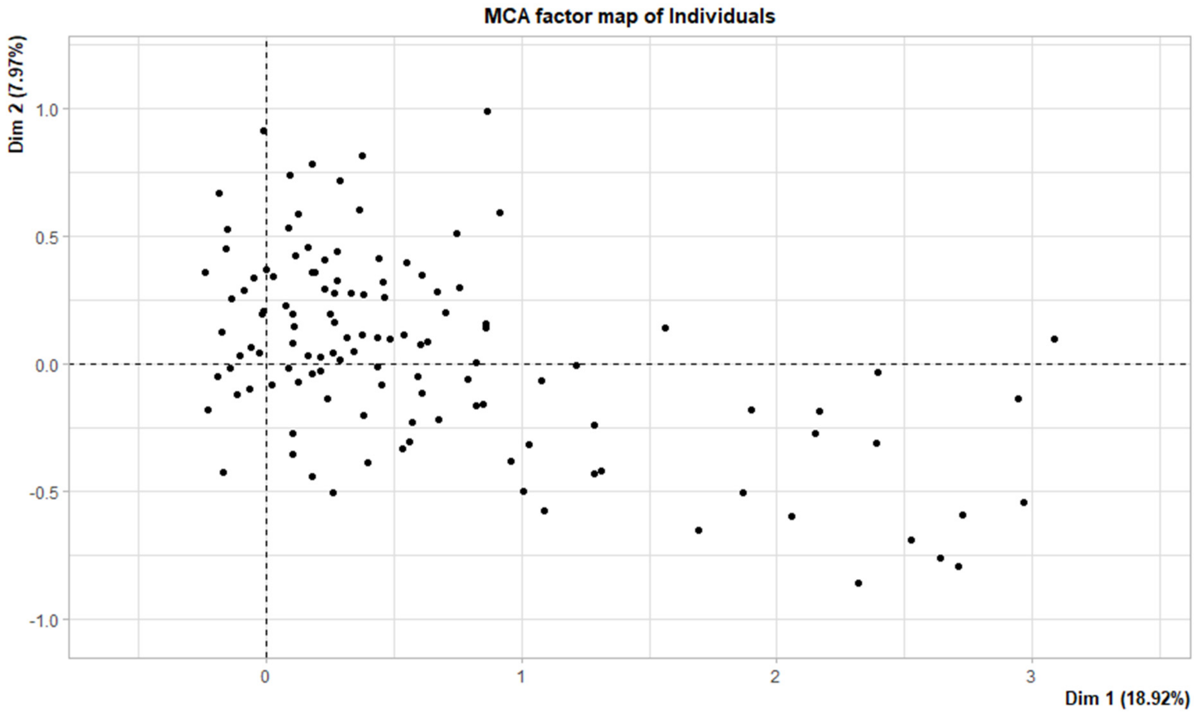

Figure S1. MCA factor map of the 514 studies captured from a scoping review on the reported causes and syndromes associated with mid to late-term pregnancy loss in horses globally.

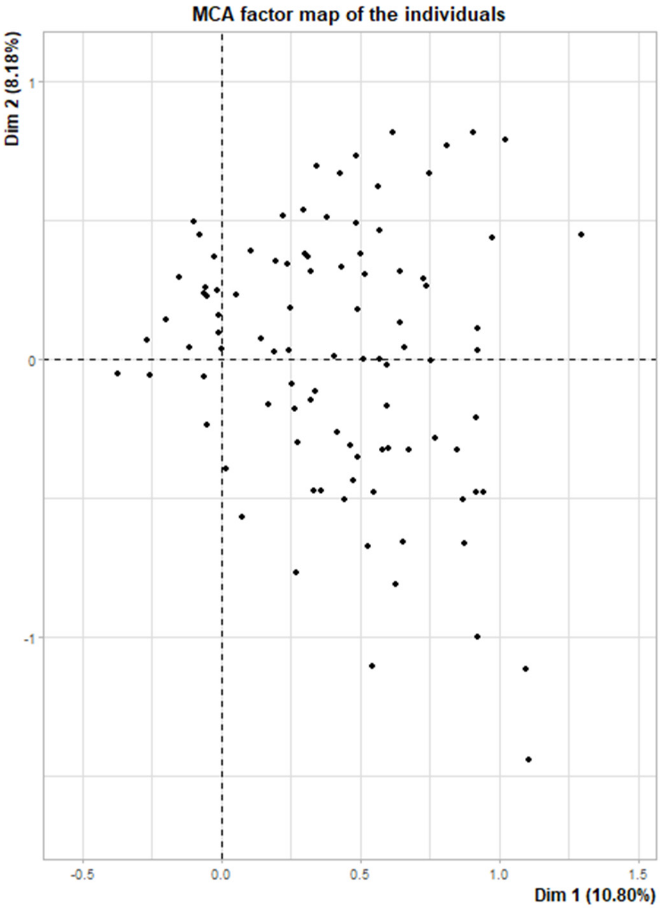

Figure S2. MCA factor map of 464 studies (dispersed records removed) captured from a scoping review on the reported causes and syndromes associated with mid to late-term pregnancy loss in horses globally

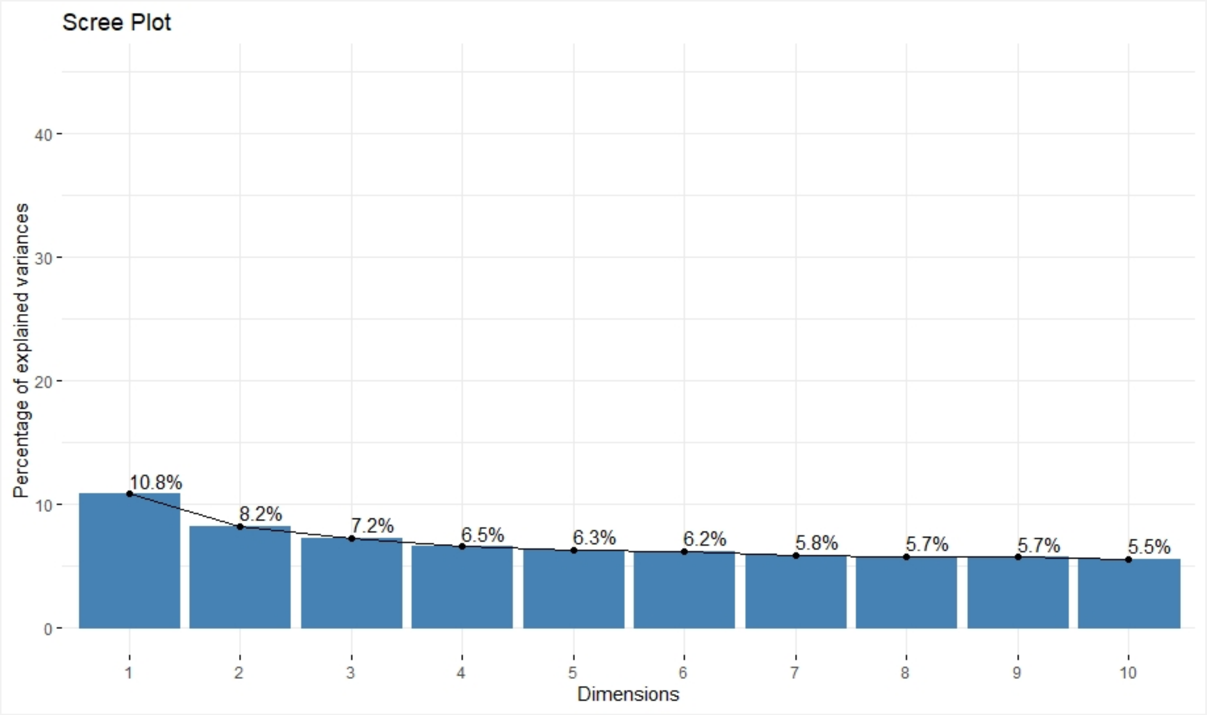

Figure S3. Decomposition of the total inertia from each dimension of the MCA.

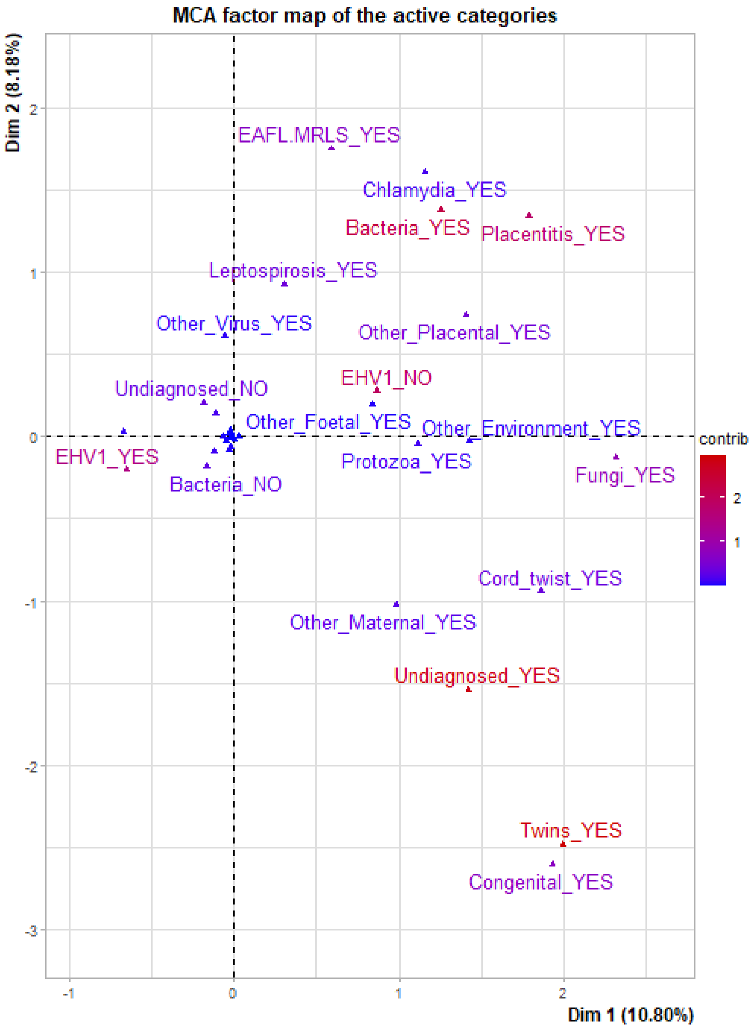

Figure S4. MCA factor map of the contribution of the active categories to dimensions 1:2.

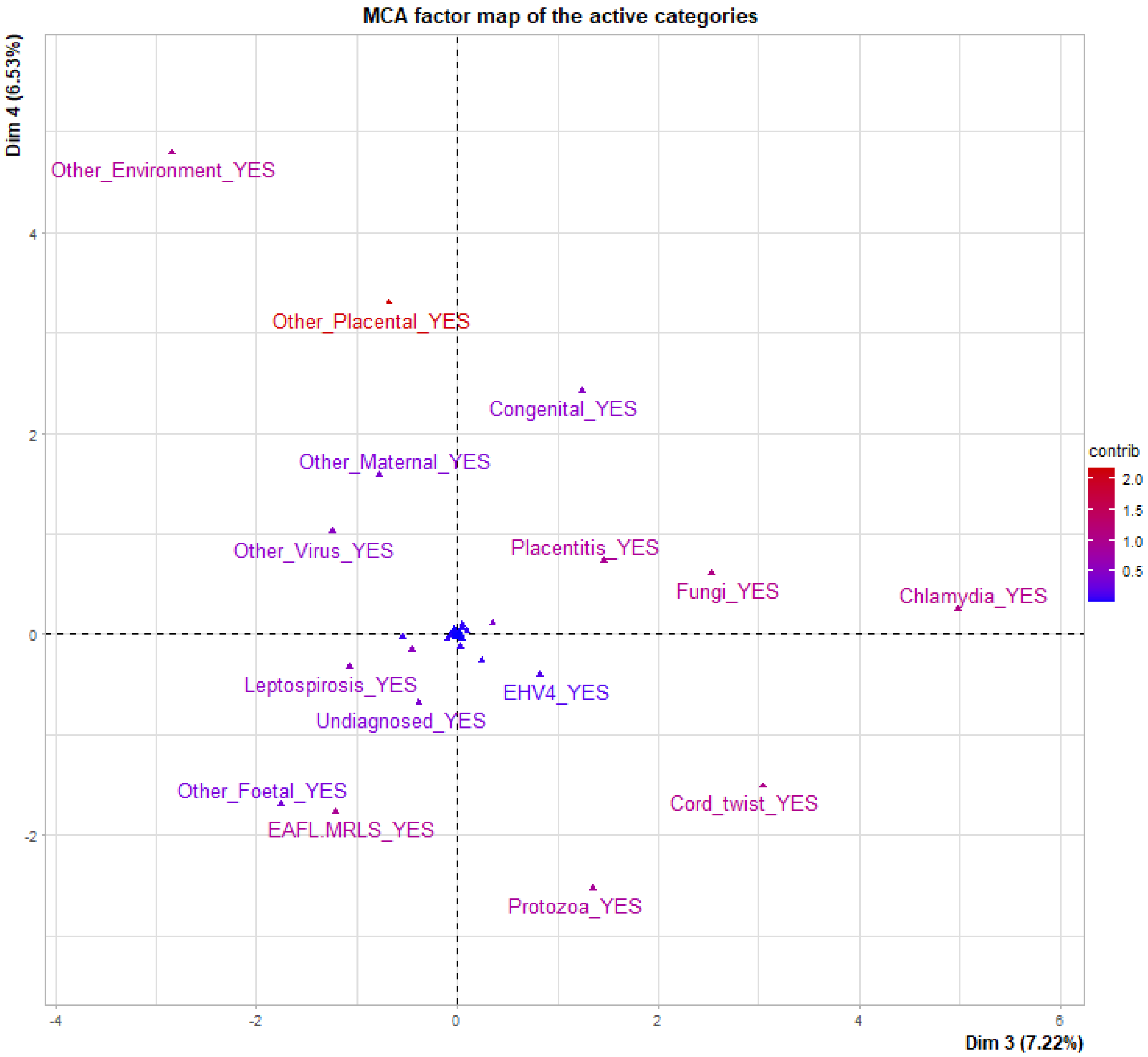

Figure S5. MCA factor map of the contribution of the active categories to dimensions 3:4.

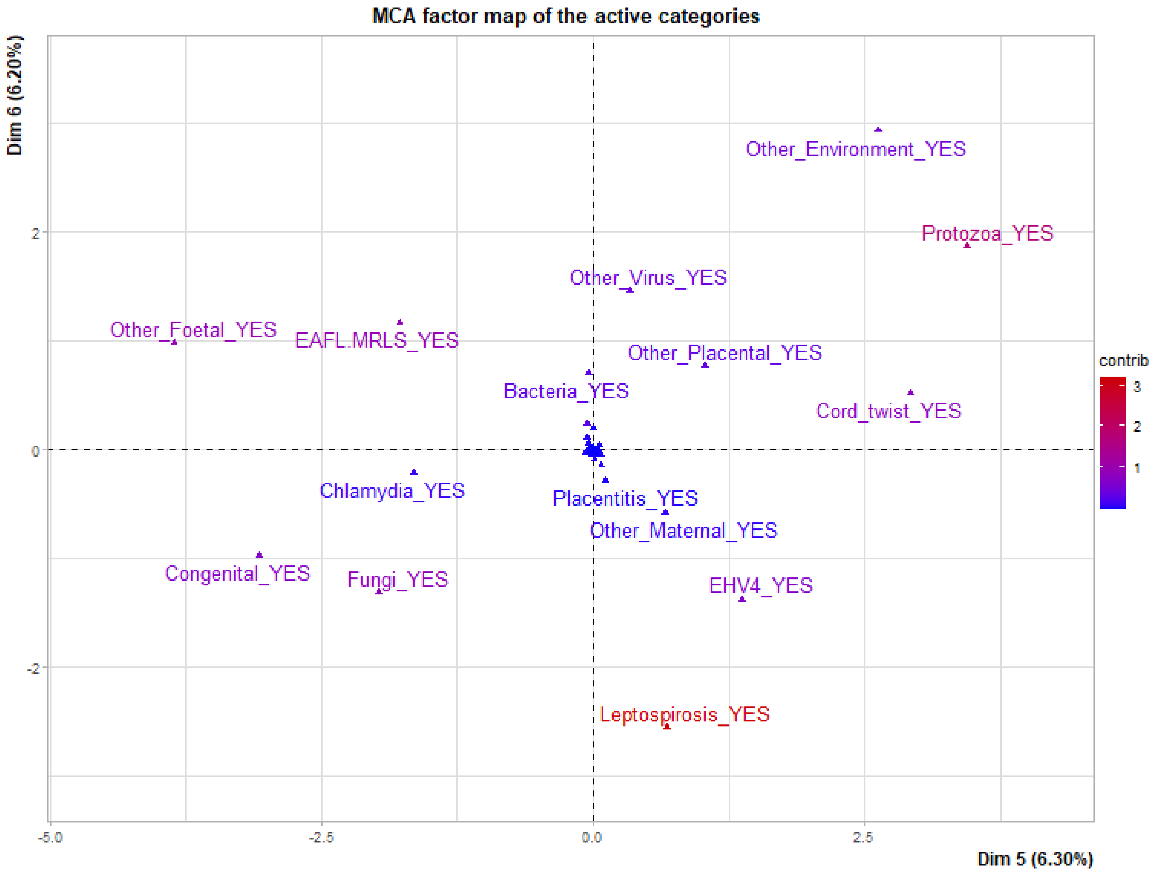

Figure S6. MCA factor map of the contribution of the active categories to dimensions 5:6.

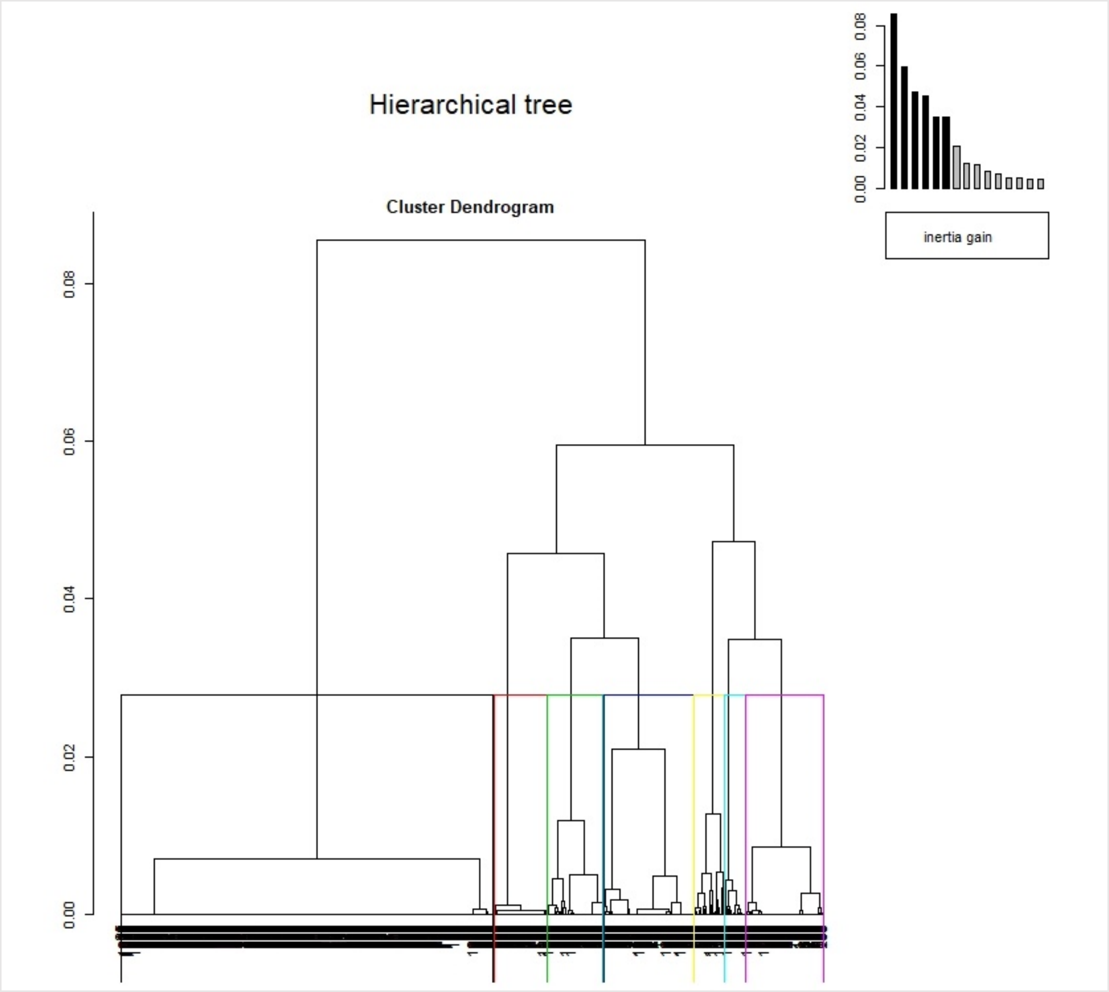

Figure S7. Hierarchical tree dendrogram and bar plot of the gain of within-inertia from the MCA.
